# The Importance of DS-14 and HADS Questionnaires in Quantifying Psychological Stress in Type 2 Diabetes Mellitus

**DOI:** 10.3390/medicina55090569

**Published:** 2019-09-05

**Authors:** Ana-Maria Pah, Petru Bucuras, Florina Buleu, Anca Tudor, Stela Iurciuc, Dana Velimirovici, Caius Glad Streian, Marius Badalica-Petrescu, Ruxandra Christodorescu, Simona Dragan

**Affiliations:** 1 Department of Cardiology, “Victor Babes” University of Medicine and Pharmacy, 300041 Timisoara, Romania; 2 Department of Internal Medicine II, “Victor Babes” University of Medicine and Pharmacy, 300041 Timisoara, Romania; 3 Department of Functional Sciences, “Victor Babes” University of Medicine and Pharmacy, 300041 Timisoara, Romania; 4 Department of Internal Medicine I, “Victor Babes” University of Medicine and Pharmacy, 300041 Timisoara, Romania

**Keywords:** type 2 diabetes mellitus, hospital anxiety and depression scale (HADS), type D personality, DS-14 questionnaire, psychological stress, risk factors

## Abstract

*Background and Objectives:* The comorbid association between type 2 diabetes mellitus (T2DM) and a psychological profile characterized by depression and/or anxiety has been reported to increase the risk of coronary heart disease (CAD), the most striking macrovascular complication of diabetes. The purpose of the present study was to quantify anxiety, depression and the presence of type D personality, and to correlate the scores obtained with cardiovascular risk factors and disease severity in diabetic patients. *Materials and methods:* The retrospective study included 169 clinically stable diabetic patients divided into two groups: group 1 without macrovascular complications (*n* = 107) and group 2 with CAD, stroke and/or peripheral vascular disease (*n* = 62). A biochemical analysis and an assessment of psychic stress by applying the Hospital Anxiety and Depression Scale (HADS)and the Type D scale (DS-14) to determine anxiety, depression and D personality scores were done in all patients. Statistical analysis was made using SPSSv17 and Microsoft Excel, non-parametric Kruskal–Wallis and Mann–Whitney tests. *Results:* Following application of the HAD questionnaire for the entire group (*n* = 169), anxiety was present in 105 patients (62.2%), and depression in 96 patients (56.8%). Group 2 showed significantly higher anxiety scores compared to group 1 (*p* = 0.014), while depression scores were not significantly different. Per entire group, analysis of DS-14 scores revealed social inhibition (SI) present in 56 patients (33%) and negative affectivity (NA) in 105 patients (62%). TheDS-14 SI score was significantly higher in group 2 compared to group 1 (*p* = 0.036). Type D personality, resulting from scores above 10 in both DS-14 parameter categories, was present in 51 patients of the study group (30%). There was a direct and significant correlation (*r* = 0.133, *p* = 0.025) between the Hospital Anxiety and Depression Scale-Anxiety (HAD-A) score and the LDL-c values. *Conclusions:* The results of this study demonstrated that more than a half of patients with diabetes had anxiety and/or depression and one third had Type D personality, sustaining that monitoring of emotional state and depression should be included in the therapeutic plan of these patients. New treatment strategies are needed to improve the well-being of diabetic patients with psychological comorbidities.

## 1. Introduction

Type 2 diabetes mellitus (T2DM) is one of the most common chronic diseases [1], with steadily rising global prevalence, that is frequently associated to a psychological profile characterized by depression and/or anxiety [2]. T2DM is characterized by an increased prevalence of death, being responsible for 4.6 million deaths each year [1].

The speedy pace of our society has created emotional instability. Negative emotional states have adverse effects on cardiovascular and other organ systems [3]. Recent epidemiological studies have confirmed that psychosocial factors are associated with an increased risk of developing coronary heart disease, which is a major cause of death and disability worldwide [4,5].

In diabetic patients, depression reduces the quality of life, which is manifested by decreased self-care and consequently a higher rate of micro and macrovascular complications, plus higher levels of HbA1c and mortality [6,7]. Also in diabetic patients with depression, health care utilization and expenditures are significantly higher than those without depression [7].

Diabetics have a two-fold risk for anxiety and depression compared to the general population [8]. Stress and depression activate the hypothalamic-pituitary-adrenal axis, stimulate the central nervous system, increase inflammation and platelet aggregation and decrease insulin sensitivity, destabilizing glycemic control and increasing the risk of complications [9,10]. A meta-analysis of 11 trials concluded that T2DM patients have a 24% greater chance to develop depression compared to non-diabetics. However, depression diagnosed prior to diabetes was taken into consideration in only one trial. Therefore, the results seem to overestimate the incidence of depression in diabetics [11].

The Maastricht study was the first cohort to address the complex association between depression and T2DM by follow-up of personality traits determined by relevant questionnaires. The study included 2861 participants. Socially isolated individuals more frequently had newly and previously diagnosed T2DM. The final analysis will allow conclusions about the mechanisms underlying the association between stress and T2DM and about prevention [12,13].

The Hospital Anxiety and Depression Scale (HADS) is a validated rapid self-assessment screening tool, [14]. Although not used for diagnostic purposes, is very convenient for self-assessment of anxiety and depression in patients with somatic or mental problems, which is very much used today. The scale sensitivity and specificity are comparable to other self-assessment screening tools [15].

Multiple biological and behavioral processes have been associated with type D personality, which predicts increased mortality and morbidity burden, and poorer health-related quality of life [16]. Type D affiliation is established by the Type D scale(DS-14), a questionnaire, which contains seven items for each of the D typology components: negative affectivity and social inhibition. Individuals with increased negative affectivity are not only dysphoric, but also have a negative image of themselves. Social inhibition is described as the tendency to avoid possible obstacles generated by social interaction, such as disapproval or lack of appreciation from others. Both negative affectivity and social inhibition are felt in the form of a hostile and unbearable social environment [17].

The purpose of the present study was to quantify anxiety, depression and presence of type D personality and to correlate the scores obtained with cardiovascular risk factors and disease severity in diabetic patients.

## 2. Materials and Methods

### 2.1. Study Population

This retrospective study was conducted between 2017 and 2018 in the Clinic of Prevention and Rehabilitation, Institute of Cardiovascular Diseases Timişoara, on 169 diabetic patients. All patients were known diabetics previously diagnosed according to the consensus report by the American Diabetes Association (ADA) and the European Association for the Study of Diabetes (EASD) in 2018based on fasting plasma glucose > 126 mg/dL or 2h plasma glucose > 200 mg/dL by oral glucose tolerance test (OGTT) [18]. The patients were divided into two groups, depending on the presence or absence of macrovascular complications. Group 1 (*n* = 107) included T2DM patients without coronary artery disease, stroke, peripheral arterial disease or any other form of cardiovascular disease (aortic aneurysm or dissection), and group 2 (*n* = 62) T2DM patients with angiographically confirmed coronary artery disease, peripheral vascular disease or previous stroke. Patients with pre-diabetes or newly diagnosed diabetes, secondary hypertension, stage III chronic kidney disease, inflammatory diseases, known infections or neoplasms were excluded from both groups. All patients maintained the previously prescribed cardiac medications at the same doses. The study was conducted with the approval of the local Ethics Commission. Informed consent was obtained in all cases. Ethical approval number 1709 (approved on 15 March 2017).

Hypertension was classified according to the most recent 2018 European Society of Cardiology/European Society of Hypertension (ESC/ESH) guidelines for management of arterial hypertension, by ambulatory and home blood pressure monitoring, as systolic blood pressure ≥135 mmHg and/or diastolic blood pressure ≥85 mmHg [19]. Abdominal obesity was defined by abdominal circumference >94 cm in men and >80 cm in women according to the International Diabetes Federation (IDF) criteria [20]. Body mass index (BMI) was determined according to the relationship weight/height^2^ (kg/m^2^), and interpreted according to the National Cholesterol Education Program, Adult Treatment Panel III [21,22]. Hyperuricemia was defined as serum uric acid (UA) ≥7mg/dL in men and ≥6mg/dL in women according to the European League Against Rheumatism Guide (EULAR) [23].

Self-administered questionnaires were used to assess history of cardiovascular disease, smoking status, diabetes medication and diabetes duration.

### 2.2. Evaluation of Biochemical Parameters

Venous blood samples were taken after 12 h of fasting for total serum cholesterol, high-density lipoprotein (HDL) cholesterol, triglycerides, low-density lipoprotein (LDL) cholesterol, glucose, HbA1c, serum creatinine and uric acid, using a Siemens Dimension RXL-MAX, Dade Behring, Erlangen, Germany. Microalbuminuria was determined in early morning urine specimens, together with the urinary albumin/creatinine ratio, using the immunoturbidimetric method and urinary creatinine by a modified kinetic Jaffe reaction (Dimension RXL-MAX, Dade Behring, Erlangen, Germany). The samples were analyzed in the hospital laboratory. Estimated glomerular filtration rate (eGFR) was calculated based on MDRD (modification of diet in renal disease) formula. eGFR = 186 × (creatinine/88.4)^−1.154^ × (Age)^−0.203^ × (0.742 if female).

### 2.3. Cardiac Evaluation

Standard transthoracic echocardiography was performed by means of a GE Vivid 9 ultrasound system, manufactured by GEMS Ultrasound, Tirat Carmel, Israel, and electrocardiographic recordings and chest X-rays were taken of all patients to certify their stable clinical condition at enrollment.

### 2.4. Assessment of Psychic Stress

Assessment of psychic stress was made by applying the HADS and DS-14 scales to determine anxiety, depression and D personality scores. Depression and anxiety were assessed using HADS [14,24]. The HADS scale is composed of 14 items and contains two subscales, one for anxiety (HAD-A) and another one for depression (HAD-D). Each item is quantified on a scale of 4 points on the Likert scale, from 0 (no symptoms) to 3 (maximum symptom level). The maximum score for each subscale is 21, scores 0–7 on each subscale are considered normal, while scores above 11 signify a considerable psychological morbidity, either anxiety or depression. Scores 8–10 indicate a borderline status. Scores were considered if at least 5 responses were given for each subscale. Missing answers in patients who completed only 5 or 6 items were replaced based on the sum of the filled items, multiplied by 7/5 respectively 7/6. The DS-14 scale has 14 items, each evaluated on a scale between 0 = false and 4 = true, also by using two subscales, one for social inhibition (DS-SI) and one for negative affectivity (DS-NA). The total score on each subscale is between 0 and 28. The use of DS-14 scale is dichotomic, requiring a score ≥10 on both subclasses to fulfil the D personality condition.

### 2.5. Statistical Analysis

Statistical processing was performed by SPSSv17 and Microsoft Excel. Numeric variables average values, standard deviations, minimum values and maximum values were estimated. The correlations between numerical variables were estimated by Pearson correlation coefficient, and the comparisons between the numerical series were performed with the non-parametric Kruskal–Wallis test for more than 2 series and with the Mann–Whitney test for comparisons between two sets of values with negative distribution. For ranked values, the medians were calculated, and the correlations between them were determined by calculating Spearman’s nonparametric correlation coefficient. For nominal variables, frequency tables were built. The associations and the comparisons between parameters were achieved by applying the chi^2^ (χ^2^) test. The estimates were considered significant for a value of *p* < 0.05.

## 3. Results

Characteristics of patients are listed in Table 1. The gender distribution of patients revealed significant differences between the two groups; the number of female patients included in group 1 was almost double than that of group 2 (63 versus 30 patients, *p* = 0.05). Significant differences were found between the two groups regarding smoking (*p* = 0.02) and TGL values (*p* = 0.04). There were no significant differences in the average age of patients. Of the total diabetic patients included in the study, only three were normotensive and the blood glucose values revealed a fairly good control of diabetes.

Diabetes-specific atherosclerotic dyslipidemia, characterized by triglycerides TG increase and HDL-c decrease, was evidenced in group 2 by the significant increase of mean TG values (192.2 ± 15.81 mg/dL) and low mean HDL-c values (38.2 ± 3.19 mg/dL), while in group 1 mean TG and HDL-c values were closer to the normal limits (154.5 ± 11.76 mg/dL and 47.8 ± 3.35 mg/dL, respectively). There was a significant difference between groups regarding diabetes duration, 5 ± 2.5 years in group 1 versus 7 ± 1.8 years (*p* = 0.02).

Kidney function was slightly altered in group 2 compared to group 1, as revealed by mean values of serum creatinine (1.27 ± 0.12 mg/dL versus1.13 ± 0.08 mg/dL, respectively), and microalbuminuria (136.78 ± 0.55 mg/dL versus 142.81 ± 0.67 mg/dL, respectively).

Obesity detected by a BMI above 30 kg/m^2^ was present in 55 patients from gr 1 (51.4%) and in 21 patients from group 2 (33.87%). In group 2, macrovascular complications were represented by stroke in four cases, peripheral arterial disease in 11 cases and coronary artery disease in 57 patients.

**Figure 1:**
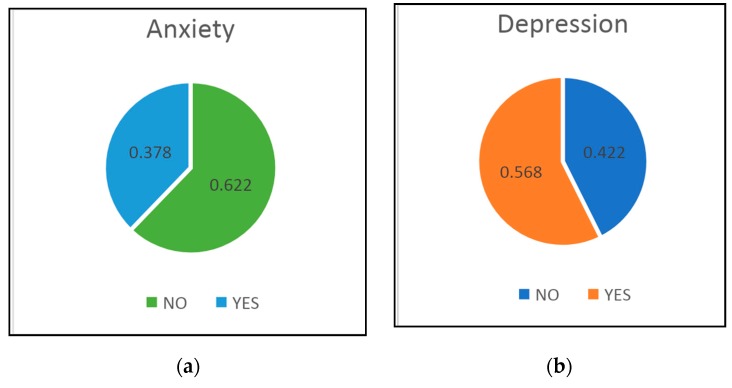
Results of applying The Hospital Anxiety and Depression Scale-Anxiety (HAD-A) levels (**a**) and The Hospital Anxiety and Depression Scale-Depression (HAD-D) levels (**b**) to the entire group (*n* = 169).

The results of applying the HAD questionnaire to the entire group (*n* = 169) are presented in Figure 1. Anxiety was present in 105 patients (62.2%), and depression in 96 patients (56.8%).

**Figure 2:**
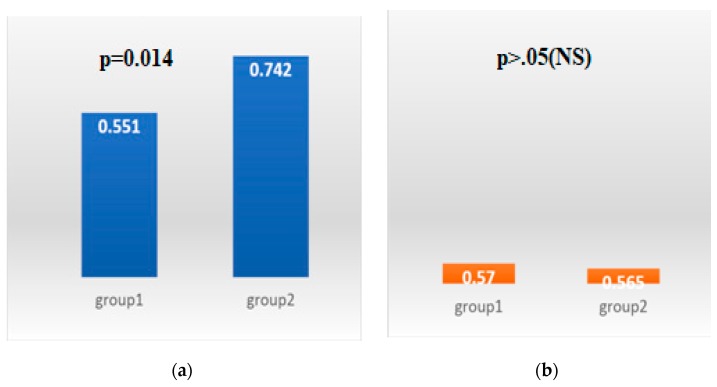
Comparison of t The Hospital Anxiety and Depression Scale-Anxiety (HAD-A) levels (**a**) and The Hospital Anxiety and Depression Scale-Depression (HAD-D) levels (**b**) in the two groups. (chi^2^ test).

Anxiety was observed in 55.1% (*n* = 59) of patients in group 1, compared to 74.2% (*n* = 46) in group 2 (*p* = 0.014), while the results of the HAD-D questionnaire did not show significant differences between the two groups (Figure 2).

**Figure 3:**
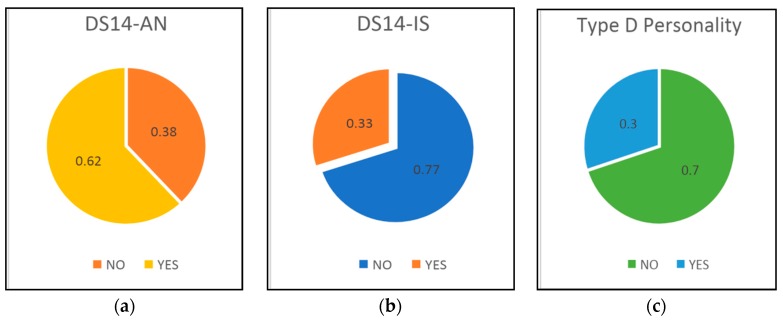
Results of applying the DS-14 questionnaire to the entire group (*n* = 169): type D scale for social inhibition (DS14-IS) (**a**); type D scale for negative affectivity (DS14-AN) (**b**) and type D personality (**c**).

The results of applying the DS-14 questionnaire to the entire group (*n* = 169) are presented in Figure 3. Social inhibition was present in 56 patients (33%) and negative affectivity in 105 patients (62%). Type D personality, resulting from scores above 10 in both Type D scale (DS-14) parameter categories, was present in 51 patients of the study group (30%).

Groups 1 and 2 were compared based on the scores of HAD-A, HAD-D, DS-14 individual scores and type D personality also by applying the non-parametric Mann–Whitney test. The comparison revealed significantly higher HAD-A scores in group 2 (*p* = 0.011) compared to group 1. TheDS-14 IS score was significantly higher in group 2 compared to group 1 (*p* = 0.036) (Table 2).

The difference between the two groups regarding type D personality was insignificant (chi^2^ test, *p* = 0.131).

**Figure 4:**
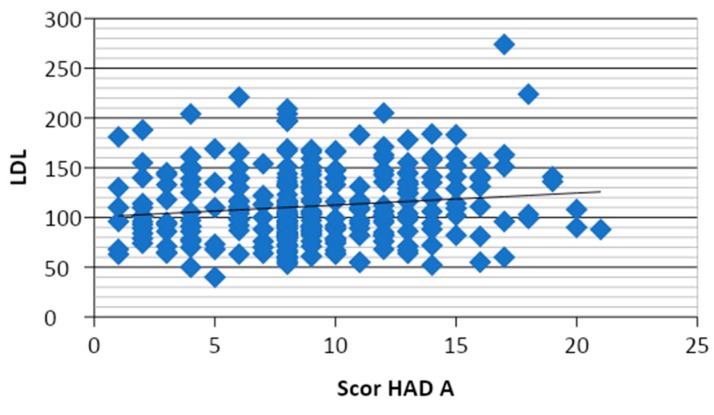
Graphic representation of the correlation between HAD-A and LDL-c score per total study patients (*n* = 169).

Of all the risk factors, only LDL-cholesterol values correlated directly and significantly (*r* = 0.133, *p* = 0.025) with the HAD-A score per total study patients (*n* = 169) (Figure 4).

The non-parametric Kruskal–Wallis test was applied to compare the psychological tests’ score results according to grades of hypertension. In group 1 the analysis revealed insignificant differences for all scores. In group 2 there were significant differences for HAD-D scores according to grades of hypertension (*p* = 0.033) (Table 3). A more refined comparison between each two consecutive grades of severity of hypertension, by applying the non-parametric Mann–Whitney test, revealed significantly higher HAD-D scores (*p* = 0.017) and DS-14 NA scores (*p* = 0.05) in grade three hypertension compared to grade two.

## 4. Discussion

Diabetes mellitus is an important risk factor for hyperlipemia and atherosclerosis, being frequently associated with hypertension, clotting disorders, increased oxidative stress, and affecting the function and anatomy of the endothelium [25]. Numerous retrospective studies have demonstrated that the relationship between depression and diabetes is bidirectional, but prospective analyses were needed in order to understand this relationship during the natural course of the disease [9]. Although there is a strong association between depression and the incidence of T2DM, the relationship is poor between T2DM and the risk of depression. T2DM is a severe metabolic disease, which causes major changes in patients’ lives. Likewise, depression is a complex disease that affects all aspects of life: social, psychological, behavioral and biological. Depression being associated to a 60% increase in the risk of T2DM, it is comparable in importance only to smoking [9,26]. In this study we found significant differences between group 1 (*n* = 32) and group 2 (*n* = 37) regarding smoking (*p* = 0.02). Anxiety was observed in 55.1% (*n* = 59) of T2DM patients without macrovascular complications (group 1) compared to 74.2% (*n* = 46) with macrovascular complications (group 2) (*p* = 0.014), while the results of the HAD-D questionnaire regarding depression did not show significant differences between the two groups. By applying the non-parametric Mann–Whitney test, we demonstrated that HAD-A scores are significantly higher in group 2 compared to group 1 (*p* = 0.011). Nefs et al. observed an increased level of depressive symptoms as frequent comorbidity in primary care of diabetic patients, with an incidence of depression occurrence in one out of four patients over 2.5 years of surveillance. New cases of diabetes in patients without previous symptoms occurred at a 14% rate. Once present, the depressive symptoms become persistent or recurrent in 2/3 of cases [27]. This observation of the recurrence of depression and its chronicity in diabetics is consistent with previous research in which about half of diabetic patients with baseline depressive symptoms continued to experience depression for 1 up to 5 years later [28]. Furthermore, 40% of diabetic patients with depressive symptoms experienced major depression in the next two years [29].

In the National Health and Nutrition Examination Survey (NHANES) conducted between 2005–2012, Wang et al. found that 49.4% of diabetic patients were taking antidepressants, which was 1.55 times more than the average of 31.8% of the US depressed population. By applying a valid questionnaire, they established increased depression scores in these patients. Depression was associated with negative impact on quality of life, affecting social relationships and even suicide. [30]. In our entire study group (*n* = 169), the observed incidence of anxiety was of 62.2% and 56.8%depression.

Khuwaja et al. have also found an increased prevalence of anxiety and depression in patients with chronic conditions, including T2DM. In their study, anxiety was present in 58% of patients with T2DMand depression in 44%. These scores are twice as high as those found in developed countries [31]. Indeed, depression and other psychological problems are more common with patients in developing countries. The explanation for this phenomenon is social instability, low levels of education, poverty, financial difficulties and gender inequality, all of which cause a high level of mental stress [32].

In a study of diabetic patients monitored for 5 years, Lin et al. demonstrated that those with depression had a clinically significant risk of micro- and macrovascular complications, compared to diabetics without depression. In severely depressed diabetics, that risk was 36% higher for advanced microvascular complications, such as terminal renal disease or blindness, and 25% higher for macrovascular complications, such as myocardial infarction (MI) or stroke [33]. In our study, from the 169 diabetic patients included, 62 presented macrovascular complications and the kidney function was slightly altered in group 2 compared to group 1, as revealed by mean values of serum creatinine and microalbuminuria. Macrovascular complications were represented by stroke in four cases, peripheral arterial disease in 11 cases and coronary artery disease in 57 patients. The results of this study demonstrated a significant correlation between HAD-A and LDL-c scores (*r* = 0.133, *p* = 0.025), and significantly increased levels of this score in patients with macrovascular complicated T2DM (Mann–Whitney nonparametric test, *p* = 0.011). Additionally, these patients had significantly increased HAD-D scores according to hypertension stages (Kruskal–Wallis nonparametric test, *p* = 0.033). A more refined comparison between each two consecutive grades of severity of hypertension, revealed significantly higher HAD-D scores (*p* = 0.017) and DS-14 NA scores (*p* =0.05) in grade 3 hypertension compared to grade 2.

The study by van Dooren et al. demonstrated an increased prevalence of both anxiety and depression symptoms in patients with T2DM. In addition, D personality type was more prevalent in T2DM, whereas the extrovert and emotionally stable type of personality was less present [34]. In the present study, the application of the DS-14 questionnaire showed the presence of social inhibition in 56 patients (33%) and negative affectivity in 105 patients (62%).We observed a significant association of the DS-14 SI score with macrovascular complications of T2DM (Mann–Whitney nonparametric test, *p* = 0.036). Type D personality, resulting from scores above 10 in both DS-14 parameter categories, was present in 51 patients of the study group (30%). The difference between the two groups regarding type D personality was insignificant (chi^2^ test, *p* = 0.131). Psychosocial, biological and lifestyle mechanisms can explain the multifactorial relationship between T2DM and mental stress. Diabetes is one of the best examples of interaction between the emotional brain and endocrine neuro-regulation for maintaining normal blood glucose levels. In order to maintain continuous stability, the human body needs to adjust the experience of immediate reality with neural and hormonal programs. These programs are a set of hierarchical feedback paths that keep memories of familiar experiences, responses and the results of some situations. New experiences activate adjustment mechanisms in an attempt to restore the previous order. When these differences are sufficiently prominent, familiarity fades away and that generates the experience of an emotion. When the adjustment mechanism fails, panic, anxiety and depression reactions occur [35]. The significant difference in the present study between groups regarding diabetes’ duration, 5 ± 2.5 years in group 1 versus 7 ± 1.8 years (*p* = 0.02), might also have contributed to further explain the increased anxiety and depression scores in T2DM patient with macrovascular complications. Therefore, for the effective treatment of diabetes, control of emotional stress is mandatory along with diet and medical treatment [36].

Gragnoli et al. believe that vulnerability to depression can be attributed to the variability of genes that regulate stress response systems in the hypothalamic-pituitary axis. Hyperactivity of the HHC axis may be caused by genetic variants of CRH receptors (CRHR1 and CRHR2), melanocortin receptors (MC1R–MC5R), the glucocorticoid receptor (NR3C1), the mineralocorticoid receptor (NR3C2) and FK506 binding protein. These variants may be partly responsible for the clinical association between depression, T2DM and MS [37].

The purpose of identifying diabetic individuals with high risks of depression is to implement preventive measures and cognitive-behavioral treatments and change their lifestyle. These preventive measures can contribute to a significant decrease in T2DM/depression comorbidity and, implicitly, the cost of treating them, which burdens the health system.

## 5. Conclusions

This study showed that more than a half of patients with diabetes had anxiety and/or depression and one third had Type D personality, demonstrating a significant correlation between these entities and T2DM with macrovascular complications. These results also sustained that the use of self-administered questionnaires for anxiety/depression and type D personality is required for the screening of diabetic and coronary patients, whether in hospital or outpatient settings. The HAD-S and DS-14 scales are short and easy to apply, the fill-in duration is only 2–5min and their accessibility allows individual completion. Clinical studies are needed in this new research field, toassociate metabolic and mental diseases. Although many retrospective studies have proved that the relationship between depression and diabetes is bidirectional, future prospective analyzes are needed to understand the development of this relationship during the natural course of the disease.

## Figures and Tables

**Table 1:** Patient characteristics at inclusion-mean ± standard deviations (SDs; *n* = 169), ns = non significant.

	Group 1 (*n* = 107)	Group 2 (*n* = 62)	*p* Value
Age (years)	63.5 ± 1.83	62.6 ± 2.37	ns
Gender (M/F)	44/63	32/30	*p* = 0.05
Smoking (Yes/No)	32/75	37/25	*p* = 0.02
BMI (kg/m^2^)	28.7 ± 4.2	27.2 ± 3.4	ns
SBP (mmHg)	150.1 ± 13.92	135.6 ± 11.71	ns
DBP (mmHg)	87.5 ± 7.35	76.3 ± 6.84	ns
Glucose (mg/dL)	141.8 ± 13.77	128.4 ± 15.22	ns
HbA1c(%)	7.54 ± 0.7	6.89 ± 0.9	ns
TC (mg/dL)	180.1 ± 15.61	161.1 ± 17.55	ns
LDL-c (mg/dL)	100.5 ± 8.27	87.9 ± 9.83	ns
HDL-c (mg/dL)	47.8 ± 3.35	38.2 ± 3.19	ns
TG (mg/dL)	154.5 ± 11.76	192.2 ± 15.81	*p* = 0.04
Uric acid(mg/dL)	5.39 ± 0.94	6.04 ± 1.15	ns
Creatinine (mg/dL)	1.13 ± 0.08	1.27 ± 0.12	ns
Microalbuminuria (mg/dL)	136.78 ± 0.55	142.81 ± 0.67	ns
Diabetes duration (years)	5 ±2.5	7 ± 1.8	*p* = 0.02

**Table 2:** Comparative analysis of HAD and DS-14 test scores in the two groups (non-parametric Mann–Whitney test), s = significant, ns = non significant.

Scale	Group	*n*	Mean	Std. Deviation	Std. Error Mean	*p*
HAD-A	Gr 2	62	8.9	4.36	0.55	0.011 ^s^
	Gr 1	107	7.0	3.83	0.37	
HAD-D	Gr 2	62	7.6	3.64	0.46	0.512 ^ns^
	Gr 1	107	7.2	3.44	0.33	
DS-14 IS	Gr 2	62	8.2	7.44	0.52	0.036 ^s^
	Gr 1	107	6.8	6.4	0.62	
DS-14 AN	Gr 2	62	14.7	9.54	1.21	0.108 ^ns^
	Gr 1	107	12.4	8.64	0.84	

**Table 3:** Comparison of psychological tests scores in group 2 (*n* = 62) according to stages of hypertension. Kruskal–Wallis nonparametric test, s = significant, ns = non significant.

Gr2 (*n* = 62)								
Variabiles	Grade HT	*n*	Mean	Std. Deviation	Std. Error	95% Confidence Interval for Mean		*p*
						Lower Bound	Upper Bound	
HAD-A	0	3	7.1	3.65	1.10	4.6	9.5	0.186 ^ns^
	1	11	8.7	5.51	3.18	−5.0	22.3	
	2	26	8.9	4.66	0.91	7.0	10.8	
	3	22	9.7	4.21	0.90	7.8	11.5	
	Total	62	8.9	4.36	0.55	7.7	10.0	
HAD-D	0	3	5.0	3.61	2.08	−4.0	14.0	0.033 ^s^
	1	11	6.8	3.10	0.61	5.5	8.0	
	2	26	7.2	4.45	1.34	4.2	10.2	
	3	22	9.1	3.49	0.74	7.5	10.6	
	Total	62	7.6	3.64	0.46	6.7	8.5	
DS-14 SI	0	3	4.3	7.51	4.33	−11.3	13.0	0.822 ^ns^
	1	11	8.2	7.59	1.49	5.2	11.3	
	2	26	9.3	7.88	1.68	5.8	12.8	
	3	22	10.5	10.13	3.06	3.7	17.4	
	Total	62	8.8	8.08	1.03	6.8	10.9	
DS-14 NA	0	3	12.5	9.10	1.79	8.8	16.1	0.050 ^s^
	1	11	13.0	13.53	7.81	−10.6	16.6	
	2	26	13.2	10.44	3.15	6.2	20.2	
	3	22	18.4	8.62	1.84	14.5	22.2	
	Total	62	14.7	9.54	1.21	12.3	17.1	
